# Empiric “Three-in-One” Bismuth Quadruple Therapy for Second-Line *Helicobacter pylori* Eradication: An Intervention Study in Southern Italy

**DOI:** 10.3390/antibiotics11010078

**Published:** 2022-01-10

**Authors:** Giuseppe Losurdo, Ilaria Lacavalla, Francesco Russo, Giuseppe Riezzo, Irene Vita Brescia, Maria Rendina, Enzo Ierardi, Alfredo Di Leo

**Affiliations:** 1 Section of Gastroenterology, Department of Emergency and Organ Transplantation, University “Aldo Moro” of Bari, 70124 Bari, Italy; ilarialacavalla@me.com (I.L.); irene.brescia93@gmail.com (I.V.B.); mariarendina@virgilio.it (M.R.); ierardi.enzo@gmail.com (E.I.); alfredo.dileo@uniba.it (A.D.L.); 2 Ph.D. Course in Organs and Tissues Transplantation and Cellular Therapies, Department of Emergency and Organ Transplantation, University “Aldo Moro” of Bari, 70124 Bari, Italy; 3 Laboratory of Nutritional Pathophysiology, National Institute of Gastroenterology “S. de Bellis” Research Hospital, 70013 Castellana Grotte, Italy; francesco.russo@irccsdebellis.it (F.R.); giuseppe.riezzo@irccsdebellis.it (G.R.)

**Keywords:** *Helicobacter pylori*, gastritis, antibiotics, eradication therapy, tetracycline

## Abstract

The eradication of *Helicobacter pylori* (*H. pylori*) may be difficult due to antibiotic resistance. Indeed, after one failure, a second-line therapy is needed and a bismuth containing quadruple therapy (BQT) with a three-in-one capsule formulation is becoming very popular. Therefore, we aimed to evaluate effectiveness and safety of BQT as a second-line therapy. We recruited consecutive patients with one therapy failure. For ten days patients received the three-in-one BQT Pylera^®^ therapy, in combination with a proton-pump inhibitor (PPI), decided at the choice of the investigator, at full dose bid. The eradication rate was calculated by intention-to-treat (ITT) and per-protocol (PP)analyses and 95% confidence intervals (CI) were calculated. Seventy-three patients were recruited, 41 females and 32 males (mean age 53.0±13.1 years). Fifty-five patients failed triple therapy with amoxicillin and clarithromycin and the remaining 18 received sequential therapy. Seventy-two patients consumed at least 90% of the capsules, while only one did not complete the therapy due to adverse events (nausea and diarrhea). By ITT analysis, BQT was successful in 62 subjects (eradication rate 84.9%, 95%CI 76.7–93.1%). By PP analysis, the eradication rate was 86.1% (95%CI 78.1–94.1%).Adverse events were observed in 14 subjects (20.5%).In conclusion, our report confirmed that BQT is effective as an empiric second-line regimen.

## 1. Introduction

*Helicobacter pylori* (*H. pylori*) is the etiologic agent of one of the most widespread infections, affecting about half the general population worldwide [1]. *H. pylori* is a Gram-negative flagellated bacterium for which the natural habitat is the human stomach [2,3], where it may engender a broad spectrum of lesions. *H. pylori* infection may sometimes be asymptomatic even if it is always associated with chronic active gastritis and, sometimes, with peptic ulcer, gastric carcinoma, and mucosa-associated lymphoid tissue lymphoma (MALT) [2,3,4]. Once diagnosed, it is important to eradicate the infection. The most used therapy involves a combination of proton pump inhibitors (PPI) and some antibiotics, such as clarithromycin, levofloxacin, amoxicillin, metronidazole, or tetracycline [1].

However, the management of *H. pylori* infection is becoming more complex, and the standard treatment is still undefined [1]. The leading cause of treatment failure is antibiotic resistance. This phenomenon has significantly increased over the past few years in many world areas [5].

A therapeutic regimen, which is not influenced by clarithromycin or dual clarithromycin and metronidazole resistance, is the bismuth quadruple therapy (BQT), also known as Pylera^®^ [6]. It consists of bismuth potassium subcitrate associated with metronidazole and tetracycline. According to recent guidelines, BQT is suggested as a first-line therapyfor geographical areas where the frequency of clarithromycin or metronidazole single or dual resistances is higher than 15% [6,7]. Triple therapy based on amoxicillin and clarithromycin is, instead, suggested only in those areas where the clarithromycin resistance is lower than 15% [6]. Therefore, the knowledge of epidemiological data concerning antibiotic resistance is of paramount importance because a regimen could be effective in onegeographical area and suboptimal in another [5].

Other factors may contribute to eradication therapy success. In detail, the bacterium is susceptible to antibiotics when the intragastric pH is 6–8, the optimal range for its replication, which occurs under the mucus layer covering the gastric mucosa [8]. 

Another factor leading to treatment failure is the nonadherence to therapy, which may be associated with personal refusal, a high daily number of pills, or physician–patient misunderstanding. This is why it is fundamental to instruct patients on infection-related disorders and the proper drug intake [9]. Even nonadherence to guidelines is a factor significantly impacting on the eradication rate [10].

Therefore, we aimed to perform a prospective, intervention study to assess the effectiveness of Pylera^®^ formulation in second-line *H. pylori* therapy.

## 2. Results

Seventy-three consecutive patients who failed a first-line regimen were recruited, 41 females and 32 males. Their mean age was 53.0 ± 13.1 years. Fifty-five patients failed the conventional 14 days of triple therapy with amoxicillin and clarithromycin and the remaining 18 received sequential therapy. Nineteen patients (26%) did not undergo endoscopy due to their young age or the absence of alarm symptoms. Upon anupper endoscopy, 3 out of 73 (4.1%) had a picture of erosive arthritis, and 36 (49.3%) showed antral hyperemia; onthe histological examination, 27 (37%) had chronic antral gastritis, 16 (21.9%) multifocal chronic gastritis, and 8 (10.9%) atrophic gastritis with metaplasia. Demographic and clinical characteristics have been reported in Table 1.

Eighteen patients received omeprazole, 15 esomeprazole, 25 pantoprazole, 10 lansoprazole and 5 rabeprazole. Seventy-two patients consumed at least 90% of the capsules, while only one did not complete the therapy due to adverse events (nausea and diarrhea). That patient failed eradication. In detail, by ITT analysis, BQT was successful in 62 out of 73 subjects, with an eradication rate of 84.9% (95% CI 76.7–93.1%). By PP analysis, the eradication rate was 86.1% (95% CI 78.1–94.1%).

**Figure 1:**
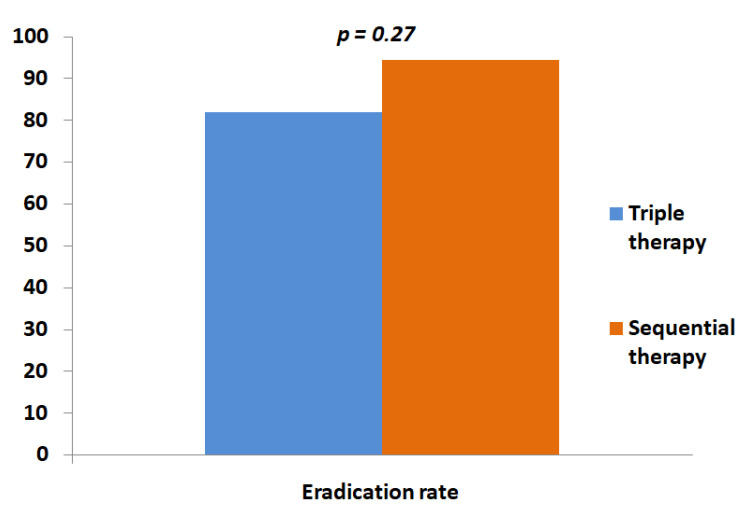
The eradication rate of BQT according to the type of first-line therapy used.

As shown in Table 2, failure was not influenced by the type of PPI (*p*=0.08) or the previously failed regimen (*p*=0.27); in detail, BQT was effective in 81.8% of those who failed triple therapy and in 94.4% of patients who failed sequential therapy (Figure 1). Finally, adverse events did not impact the eradication results (*p*=0.39).

Adverse events were observed in 14 subjects (20.5%). The most common ones were diarrhea (six patients) and weakness (six patients). Nausea (two patients), dysgeusia (two patients), and headache (two patients) were recorded less frequently. Five patients experienced more than one symptom.

## 3. Discussion

BQT is currently one of the most used eradication regimens for *H. pylori*, both in first-line and rescue therapy. In our group of patients, we demonstrated eradication of 84.9% when used as the second-line therapy. This result agrees with other reports from Italy. For example, Fiorini et al. [11] showed a success of 81%, while Zagari et al. [12] found an eradication rate of 87.5% in a multicenter study. A large study from the European Registry on *Helicobacter pylori* demonstrated the effectiveness of 89% in 375 patients in second-line therapy [13]. Even in other regions, such as Korea, the treatment was proven effective with a similar eradication rate. Indeed, BQT is one of the options suggested by guidelines after one failure [14]. Bismuth plays a relevant role as an adjunctive agent for bacterial eradication, since it is able to downregulate virulence proteins such as CagA and VacA, disturb flagella assembly (which are responsible for bacterial colonization) and inhibit antioxidant enzymes, including catalase, catalase-related peroxidase, and superoxide dismutase [15]. In combination with tetracycline-based regimens, bismuth may reach eradication rates higher than 80%, even in a third-line setting [16]. The beneficial role of bismuth in eradication regimens [17,18] has been underlined by a meta-analysis of seven randomized clinical trials, showing an odds ratio of 2.81 when bismuth was added to standard regimens [19].

Among other conventional second-line regimens, the levofloxacin-based triple therapy is, if BQT lasts 10 or 14 days, equally effective, as outlined by a meta-analysis [20]. Consequently, Canadian guidelines have suggested extending the duration of BQT to 14 days in order to optimize eradication [21]. Nevertheless, the present study still proves that a 10-day regimen is effective as a second-line treatment in our area.

The sub-analysis of eradication based on the previous first-line regimen confirmed that BQT is effective independently from the previous regimen, and it should be considered after an unsuccessful therapy containing clarithromycin. This finding confirms an observation that has already been proven in the literature [22,23].

One of the concerns about the three-in-one formulation of BQT is the compliance of the patient. This regimen consists of 12 pills to be consumed daily, which is much more than the usual regimens. However, in our experience, most patients were able to consume >90% of the pills. This finding agrees with previous evidence. It may be the consequence ofa proper explanation of the consumption mode to the patient, improving motivation and compliance, thus enabling better outcomes [24].

Finally, treatment-related adverse events are another critical topic about BQT, since the high dose of metronidazole and the association with tetracycline are considered a major cause of patient intolerance. In our research, we recorded side effects in 20.5% and only one early interruption. This finding is even better than that reported in the literature, with published rates ranging from 30% to 60% [12,25]. Coadministration of probiotics could both improve tolerability and improve eradication in this context [26,27,28,29].

The present study has some limitations. The most important is that it is an open study; therefore, BQT was not compared to another standard rescue therapy. However, considering that failure is not so common, partitioning a small number of patients into two groups would have reduced the sample size of each arm and the power of the study. Second, physicians were allowed to choose the PPI freely. Different PPIs could have engendered heterogeneity, but the analysis reported in Table 2 elucidated that BQT effectiveness is not influenced by the type of PPI [12,30]. Despite our paper being similar to other studies in the literature, it is important to underline that the results of *H. pylori* eradication are strongly dependent on a “geographic factor”, i.e., the variability of the response according to different geographic distributions of antibiotic resistance [5]. Moreover, the changes in time and trends of antibiotic resistance may cause a decline in therapy effectiveness, therefore new studies that update the panorama of BQT are always important [31].

In conclusion, our report confirmed that BQT is effective as an empiric second-line regimen. Therefore, its use may be recommended, but it should be adopted with caution since inappropriate use could undermine its effectiveness, taking into account the risk of tetracycline resistance spreading [32,33].

## 4. Materials and Methods

### 4.1. Patients

The present study was a prospective, nonrandomized, open study. Consecutive patients who failed a first-line regimen were recruited in the period January 2017–2020. Patients received for ten days the three-in-one BQT Pylera^®^ therapy, in combination with a proton-pump inhibitor (PPI), decided at the choice of the investigator, at full dose bid, thirty min before a meal (pantoprazole 40 mg bid or lansoprazole 30 mg bid or omeprazole 20 mg bid or esomeprazole 40 mg bid or rabeprazole 20 mg bid). Each capsule of Pylera^®^ contains metronidazole 125 mg, tetracycline 125 mg, and bismuth potassium subcitrate 140 mg, and patients received three pills four times a day after a meal.

Exclusion criteria were: an age < 18 years, unwillingness to participate in the study or express valid consent, use of antibiotics in the month before the inclusion or the post-treatment test, use of PPI in the two weeks before the eradication test, and gastric cancer.

For each patient, we recorded the following data: age, sex, type of PPI consumed, previous first-line eradication regimen, and upper endoscopy/gastric histology pattern if endoscopy was indicated according to the current guidelines. Additionally, we recorded adverse events and compliance by a personal interview about one week after the treatment. Good compliance was defined as taking at least 90% of the prescribed pills.

The study was approved by the Ethics committee of the AOU Policlinico Consorziale di Bari (protocol no.0064737).

### 4.2. H. pylori Investigation

The diagnosis of *H. pylori* infection was accomplished if the urea breath test was still positive, six weeks after first-line therapy completion. If the patient had an indication to perform esophagogastroduodenoscopy, at least two biopsy samples were taken from the corpus and two from the antrum.

*H. pylori* eradication was evaluated at least six weeks after the end of treatment by urea breath test, and treatment was deemed successful if the urea breath test was negative.

### 4.3. Statistical Analysis

Continuous variables were expressed as mean with standard deviation (SD) and compared by the Mann–Whitney test. For percentages/proportion, the 95% confidence interval (CI) was calculated, and Fisher’s exact test was used to compare dichotomous variables

The eradication rate of *H. pylori* was estimated both byintention-to-treat (ITT) and per-protocol (PP) statistical analyses. All tests were two-tailed, and a *p*-value < 0.05 was considered statistically significant. All analyses were performed using Graphpad Prism version five statistical software for Windows (San Diego, CA, USA).

## Figures and Tables

**Table 1:** Main demographic and clinical features of enrolled patients.

Variable (*n* = 73)	Mean ± SD, or *n* (%)
Female/male ratio	41/32
Age (years)	53.0 ± 13.1
Symptoms Dyspepsia Heartburn Regurgitation Post-prandial fullness Epigastric pain Asymptomatic	45 (61.6%) 26 (35.6%) 39 (53.4%) 12 (16.4%) 17 (23.3%) 14 (19.2%)
Endoscopic and histological picture (n = 54) Erosive arthritis Antral hyperemia Chronic antral gastritis Multifocal chronic gastritis Atrophic gastritis with metaplasia	3 (5.5%) 36 (66.7%) 27 (50%) 16 (29.6%) 8 (14.8%)
Previous failed regimen Triple therapy Sequential therapy	55 (75.3%) 18 (24.7%)

**Table 2:** Comparison between patients who failed BQT versus successful therapy.

Variable	Success (*n* = 62)	Failure (*n* = 11)	*p*
Age	51.6 ± 13.3	59.5 ± 9.4	0.07
Male sex	25 (40.3%)	7 (63.6%)	0.19
PPI Omeprazole Esomeprazole Pantoprazole Lansoprazole Rabeprazole	17 (27.4%) 10 (16.1%) 20 (32.2%) 10 (16.1%) 5 (8.2%)	1 (9.2%) 5 (45.4%) 5 (45.4%) 0 (0%) 0 (0%)	0.08
First line Triple Sequential	45 (72.6%) 17 (27.4%)	10 (90.9%) 1 (9.1%)	0.27
Adverse events	11 (17.7%)	3 (37.3%)	0.39

## Data Availability

Not applicable.
